# Medical Opinion and Movement

**Published:** 1908-06-20

**Authors:** 


### Medical Opinion and Movement.

Since the much discussed visit of Lorenz to this country a few years ago, the subject of congenital dislocation of the hip has receded somewhat into the background. A new procedure for dealing with this trouble without operation is put forward by Dr. Hibbs, of New York. The principles upon which his treatment is based are briefly these. By adducting and flexing the thigh with the knee extended, it is possible to force the head of the femur below and behind the acetabulum. To compel the head to travel upward and forward when the thigh is extended and brought back to the middle line, the patient's pelvis is firmly strapped to a wide board fitted with openings beneath the trochanters, in which are pads which can be screwed upwards so as to force those bony prominences forwards. The result is that by adjusting these pads while the thigh is still flexed and adducted, the dislocation can be reduced; plaster is then applied with the thigh abducted and the knee extended, so as to keep the hamstrings taut. Every two weeks the plaster splinting is to be removed, and it can each time be reapplied with the limbs nearer their normal position; and it is claimed that the results are highly successful. Moreover, according to the inventor, the method is applicable to children considerably older than Lorenz's maximum age limit, and the cure is complete in two or three months. Details are given in the *New York Medical Journal* of fourteen successful cases in which the ages of the children varied from twenty-one months to eleven years.

---

Even among the most authoritative of our midwifery text-books there is a certain amount of disagreement over the estimation of the probable date of labour. Some obstetricians reckon 280 days onwards from the first day of the last normal menstruation, while others add the same number from the last day; others take the figure as 273 days from the end of menstruation, a method very similar to that first mentioned. Dr. Brodhead, of New York, discusses the whole subject in the *Post-Graduate* on an analysis of 500 cases. He concludes that the date of menstruation is the surest guide, but finds that 45 per cent. of his patients have no accurate knowledge of the exact date of their last period; of those who have, less than a third are confined within four days of the expected date, reckoning on Nægele's principle of adding seven days to the first day of the last period, and counting three months back; but 76 per cent. are delivered within a fortnight of the estimated time. Most of the other methods that have been advocated he regards as much less trustworthy, though one or two are valuable quite at the end of pregnancy, such as the engagement of the head in the brim in primiparæ, which is the rule during the last week, and the condition of the cervix as regards softening and secretion. Quickening, as might be expected, he regards as very little to be relied on, but the height of the fundus above the pelvic brim affords information which is quite valuable as a confirmation of the deductions from menstrual history. At six months he finds the fundus at the level of the umbilicus, a level (if calendar months are meant) rather lower than that commonly assigned to it by British observers at that period, and the other levels are proportional. A certain amount of the confusion that still surrounds this question will never be cleared up while authors omit to state whether their words refer to lunar or to calendar months, and we do not gather from Dr. Brodhead's remarks in which sense he uses the term.

---

At a recent meeting of the Société Clinique des Hôpitaux de Bruxelles, Dr. Wybauw read an interesting communication upon a new $x$-ray apparatus, which he claims will render great service in the examination of cases of cardiac disease. The rays of the ordinary apparatus are divergent, so that a distorted picture of the object looked at is thrown upon the screen. The new apparatus overcomes this drawback by causing parallel rays to traverse the object, with the result that the shadow thrown on the screen gives a very accurate idea of the actual size of the object. As the result of numerous observations upon all classes of subjects, Wybauw is led to the conclusion that the apparatus is of great use in the treatment of women suffering from ptoses after confinement, or corpulent individuals, and even of subjects undergoing hydro-therapeutic treatment in consequence of a heart lesion. As a means of clinching a diagnosis arrived at by the ordinary methods of percussion, the apparatus is invaluable—even small variations of cardiac volume are easily recognised by its help. By means of this method of orthoradiography it has been found that the right border of the heart varies its position with the attitude of the patient. When the heart is vertical the limits of its right border attain to, and sometimes pass beyond, the right border of the sternum.

---

We have so little exact knowledge of the factors which determine an attack of hay fever that a recent observation of Dr. Rauge (of Challes) is of interest in calling attention to the influence of altitude in its ætiology. Dr. Rauge travelled with a patient suffering from the disease in its most virulent form, and noticed that when the patient was at a high altitude his symptoms rapidly disappeared, only to reappear again as soon as a lower level had been reached. Various possible explanations suggest themselves for the reaction of this patient to different heights above the sea-level. Those who believe that hay fever is in the main a neurosis will not be inclined to pay too much attention to altitude as a factor in its causation. On the other hand, there are some who will maintain that the most probable explanation of the behaviour of Dr. Rauge's patient lies not in differences of atmospheric pressure at different levels, nor in differences of temperature or purity of the air; but in the changes in the character of the vegetation which are usually associated with the ascent of mountains. Thus on the supposition that hay-fever is directly due to the influence upon susceptible people of toxic proteid substances in the pollen of certain grasses, the simplest solution of the matter is to be sought for in the absence of these particular grasses at high levels. Indeed, previous authorities have recommended residence on mountain tops (where vegetation is scanty) as a prophylactic against hay-fever during the pollen season.

---

Synovitis set up by some injury or by an inflammatory attack of a rheumatic nature is frequently followed by a condition of pain and disability of the joint without any definite evidence of morbid changes in the joint structures. Sometimes the synovitis leaves a plastic exudate which forms adhesions within the joint. By gradual contraction these adhesions limit the range of movement of the joint and cause pain and tenderness on attempts at motion. These cases often clear up completely by careful manipulation and massage, but not infrequently adhesions have engaged so large a part of the synovial structures that only a small secreting surface is left. So-called "dry joints," in which the morbid condition appears to consist essentially in a lack of synovial fluid for lubrication, most commonly follow attacks of rheumatic inflammation. For these conditions Professor R. T. Morris, of New York, has devised an artificial lubricant which he injects into the joint cavity. The lubricant consists of one part of boroglyceride, three parts of glycerine, and four parts of normal saline solution. The solution is made upon the spot, sterilised by heat, and injected with a needle sufficiently large to carry the syrupy fluid. The author has tried these injections in several cases of painful creaking joints with adhesions, and has obtained prompt relief, which remains, at any rate, for some months afterwards. He has even risked these injections in three cases of adhesions following cured tuberculosis, and has obtained satisfactory results in all.

---

Some interesting cases are reported by Professor Max Einhorn, of New York, illustrating the differential diagnosis between cholelithiasis and certain affections of the stomach, notably benign ischochymia. It is generally recognised that cholelithiasis does not always run a typical course, and may resemble in symptoms some morbid gastric condition—hyperchlorhydria, ulcer. The pain may be diffuse, and the determining signs of icterus and enlarged liver may be absent. On the other hand, as Professor Einhorn points out, it is not so well recognised that cases of disease of the stomach, especially ischochymia, may present the typical picture of gall-stone colic. One case related by the author is that of a woman, aged forty-three, who had suffered for some time from periodical attacks of pain in the epigastrium, accompanied by vomiting, and lasting several days. The diagnosis of gall-stones was made by several competent medical men. Careful examination, however, showed no enlargement of the liver, but a much-dilated stomach. Gastric lavage in a fasting condition always showed the presence of food remnants of the day previous. As the condition did not yield to treatment, an operation was performed. A much-narrowed pylorus was found and a normal gall-bladder. Gastro-enterostomy was performed with satisfactory results. Gastric lavage is an important aid in the diagnosis of these conditions, showing the presence of food remnants from the day previous. Moreover, several of the cases of benign ischochymia cited by Dr. Einhorn have yielded to this form of treatment without resort to surgical measures.

---

An interesting discussion took place at a recent meeting of the Medical Society in Vienna upon a case of optic neuritis associated with pregnancy, introduced by Dr. Meissner. Six years previously the patient had syphilis with optic neuritis, which yielded to specific treatment. Subsequently she had four children, and with each pregnancy her eyes became affected again. She was now three months advanced in her fifth pregnancy, and had applied to the hospital to have premature labour induced in order to save her eyesight, which was now much worse again. The presence of optic neuritis was confirmed by examination, and also a diminution of the field of vision. Specific treatment had not improved the condition this time, and the question was how far pregnancy might influence the condition, and whether it was justifiable to induce premature labour. It was decided eventually to await further developments, but in the course of the discussion Professor Hochenegg made the interesting observation that he had seen one case of a woman whose hypophysis cerebri increased in size during pregnancy, causing optic neuritis by pressure. The condition subsided after confinement, but left a weakened eyesight. Professor Reuss recorded a similar case of a woman who gave birth to sixteen children. She suffered from bad sight during each pregnancy, but the condition subsequently remained stationary, and when she died, aged seventy-one, she was not blind.

---

The recent advances in the physiology of the glands all tend to demonstrate that for the most part they possess a double function: that in addition to their recognised external secretion, they produce an internal secretion which in some way or another influences the general metabolism of the body. Apart from the recognised close relationship between the mammary glands and the genital organs, presumably by some internal secretion, there is also considerable evidence in support of a similar connection between these glands and the thyroid. Atrophy of the breasts has been frequently recorded in cases of exophthalmic goître, and is considered by Boin a very usual early indication of this disease. MM. Sainton and Fernet, in discussing the question, put forward the hypothesis that this atrophy is due to the excessive activity of the thyroid, that the mammary and thyroid are in a measure complemental to each other. In support of this theory are instanced cases of myxœdema, in which the insufficiency of the thyroid is accompanied by a hypertrophy of the breasts. Djemil Pacha has recorded a case of a young man who developed painful hypertrophy of both breasts, resembling those of a pregnant woman, with secretion of a fluid similar to milk. Removal of the breasts was followed by a typical condition of myxœdema, to which he rapidly succumbed, in spite of thyroid medication. Administration of thyroid has been found to increase lactation, and the elimination of thyroidine by the milk has been noted by several observers. This function of the glands and their internal secretions is undoubtedly a most important as well as a highly interesting subject, and this particular aspect of the question is worthy of further study and research.

---

THE interesting communication of Professor Trendelenburg, of Leipsic, regarding his new operation for embolism of the pulmonary artery, to which we referred in a recent number of The Hospital, has stimulated other surgeons, and it is gratifying to record that the new operation has been attempted with success not many days after the original communication was made to the Berlin Surgical Congress. The operator was Dr. Sievers, one of Trendelenburg's assistants at the Leipsic surgical clinic; and the operation was done under very difficult conditions. The patient, a middle-aged woman, was brought to the ward with a diagnosis of appendicitis, but her condition, on examination, was such as to justify expectant treatment. Ten days after admission she was suddenly seized with symptoms pointing to pulmonary obstruction. "She presented," says Dr. Sievers, "the typical picture of a case of pulmonary embolism, and as she was practically moribund it was decided to operate." The operation, which was done at half-past five in the morning, was entirely successful. Two large clots were removed from the orifice of the pulmonary vessels, and it is interesting to note that "the moment the first clot was detached by the forceps the patient gave a gasp and respiratory movements recommenced." The wound was sewn up with catgut sutures and the patient put back to bed. Notwithstanding every effort, her condition did not improve, and she died twelve hours after the operation. This case must be regarded as the first successful attempt to remove an embolus from the pulmonary vessels—successful in so far that it restored an apparently moribund patient, although it failed to prevent the collapse, which led to a fatal result twelve hours later. It has shown, as Dr. Sievers justly remarks, that the operation is practicable and that it is justifiable in certain cases.

---

IN 1887 Hirschsprung published the report of two cases of idiopathic dilatation of the colon, and since then many cases of this "new disease" have been published both here, in America, and on the Continent. So little is known about its pathology and its treatment has hitherto been so unsatisfactory that new cases published must be interesting. The two most recent cases reported are by Professor Kohts, of Strassburg, and in both the patients were children, boys aged three and six years respectively. In the first case there was a history of abdominal distension, constipation, and gastro-intestinal trouble almost since birth; from the fifth month the child had to receive purgatives. In the second case the distension and intestinal trouble dated back four years. In both cases the children complained of colicky pains, and in both cases slight improvement followed the daily injection of oil enemata and the enforcement of an easily digestible diet combined, with abdominal massage. No permanent progress was made in either case, and both children died, as seems to be usual in such cases, suddenly. The author thinks that early and persevering treatment on similar lines hold out the best hope. Surgical interference has so far not been very encouraging. Bossowski, of Cracow, has resected the dilated colon in a boy of seven, and sutured the two ends of the intestine directly, and the result was excellent. The boy, however, was lost sight of, and the further progress of the case is not known. In a series of 41 cases analysed by Biedert, 23 died, 11 were lost sight of, and only 2 could be regarded as cured.

---

JENISSEN recently reported on an interesting case of visceral syphilis. The patient, a thirty-three-year-old soldier who had had a primary chancre, was admitted for œdema of the lower extremities and slight ascites, without fever or pain. Liver and spleen were palpable, but patient had never had malaria, and repeated examinations for the parasite were negative. There was a slight rise of temperature daily, the evening temperature being less than a degree higher than the morning temperature. A month after admission patient began to complain of gastro-intestinal symptoms, colicky pains, vomiting, and nausea. These occurred in paroxysms during which patient was collapsed. The urine contained no albumen, but there was a much increased alimentary glycosuria. As patient was getting worse, and the diagnosis was not certain, an exploratory laparotomy was decided upon. At the operation the abdomen was found full of dark-coloured fluid; the intestines themselves appeared dark in colour; there was no cirrhosis of the liver; the spleen, kidneys, stomach, and pancreas were normal; and the only abnormality found, beside the free fluid in the peritoneum, was a large number of slightly enlarged lymph glands. The abdomen was closed, and the patient was put on antisyphilitic treatment, under which he rapidly recovered.

---

AN interesting contribution to the literature of nephritis is the paper of Callisto in a recent number of the *Gaz. degli Ospedali*. The author has made a series of observations on patients suffering from interstitial nephritis, and comes to the conclusion that kidney lesions are definitely amenable to systematic massage. The massage may be done in various ways—by massaging the kidney region directly or by making certain body movements which have for their object the alteration in situation of the movable abdominal viscera. Experiments made on animals showed that lumbar massage directly increases the blood flow to the kidneys, and led to an increased elimination of urine, and that this eliminated urine was not a mere polyuria, but that the solid constituents were definitely increased. The author thinks that the massage treatment is worthy of some attention in the future, and recommends that it should be tried in selected cases.

